# Bimanual Force Coordination in Parkinson’s Disease Patients with Bilateral Subthalamic Deep Brain Stimulation

**DOI:** 10.1371/journal.pone.0078934

**Published:** 2013-11-11

**Authors:** Stacey L. Gorniak, Cameron C. McIntyre, Jay L. Alberts

**Affiliations:** 1 Department of Health and Human Performance, University of Houston, Houston, Texas, United States of America; 2 Centers for Neuromotor and Biomechanics Research and Neuro-Engineering and Cognitive Science, University of Houston, Houston, Texas, United States of America; 3 Department of Biomedical Engineering and Center for Neurological Restoration, Cleveland Clinic, Cleveland, Ohio, United States of America; 4 Cleveland Functional Electrical Stimulation Center, Louis Stokes Veterans Affairs Medical Center, Cleveland, Ohio, United States of America; University of Medicine & Dentistry of NJ - New Jersey Medical School, United States of America

## Abstract

**Objective:**

Studies of bimanual actions similar to activities of daily living (ADLs) are currently lacking in evaluating fine motor control in Parkinson’s disease patients implanted with bilateral subthalamic deep brain stimulators. We investigated basic time and force characteristics of a bimanual task that resembles performance of ADLs in a group of bilateral subthalamic deep brain stimulation (DBS) patients.

**Methods:**

Patients were evaluated in three different DBS parameter conditions off stimulation, on clinically derived stimulation parameters, and on settings derived from a patient-specific computational model. Model-based parameters were computed as a means to minimize spread of current to non-motor regions of the subthalamic nucleus via Cicerone Deep Brain Stimulation software. Patients were evaluated off parkinsonian medications in each stimulation condition.

**Results:**

The data indicate that DBS parameter state does not affect most aspects of fine motor control in ADL-like tasks; however, features such as increased grip force and grip symmetry varied with the stimulation state. In the absence of DBS parameters, patients exhibited significant grip force asymmetry. Overall UPDRS-III and UPDRS-III scores associated with hand function were lower while patients were experiencing clinically-derived or model-based parameters, as compared to the off-stimulation condition.

**Conclusion:**

While bilateral subthalamic DBS has been shown to alleviate gross motor dysfunction, our results indicate that DBS may not provide the same magnitude of benefit to fine motor coordination.

## Introduction

Bilateral deep brain stimulation (DBS) of the subthalamic region has been identified as an effective procedure for the treatment of cardinal motor signs in advanced Parkinson’s disease (PD) [1]. Improvements in motor function following DBS implantation have been reported in clinical and laboratory-based outcomes. In clinical evaluations, Unified Parkinson’s disease Rating Scale motor scores (UPDRS-III) have decreased 40–65 percent when treated with DBS [2]–[4]. Similarly, objectively measured (laboratory-based) motor control outcomes have also been shown to improve following DBS [5]–[8]. Evidence of improvement in the control and coordination of grasping forces of goal-directed upper extremity function has been reported with DBS [5], [9]–[11].

Despite such reports, few have ventured beyond evaluation via the UPDRS-III and basic features of unimanual prehension [12]–[16]. Assessment of motor dysfunction in activities of daily living (ADLs) is the next step in determining the extent of fine motor deficits with PD, as well as identifying how treatments, such as DBS, may alleviate impairment. Assessment of fine motor function has been recognized as a means to quantify subtle motor behavior deficits during PD progression [17], [18]. Impaired coordination of small motor units, unusually large grip forces, abnormal grip force production rates, and difficulty in isometric torque control have already been detected in medically managed PD [12]–[16], [18], [19].

Given that motor symptoms, medical side effects, and general quality of life worsen with PD progression [20], [21], some patients choose to undergo DBS implantation as a means to improve quality of life. Following DBS implantation surgery, stimulation parameters that best alleviate patient motor symptoms are programmed for each implanted device [3], [22]. This process of parameter programming and fine-tuning is subjective, guided by clinician intuition and experience [22]. Additional subjectivity is introduced into the programming process as select items of the UPDRS rating scale are utilized to evaluate motor function [23].

While clinically determined stimulation parameters have been effective in improving overall motor function in uni- and bilaterally implanted PD patients [2], [11], declines in cognitive function have been associated with DBS [24], [25]. As a means to mitigate DBS-induced cognitive dysfunction during clinical programming, computational software tools have been developed to optimize both cognitive and motor performance in DBS implanted patients [22], [26], [27]. Recently, these model derived stimulation parameters have been found to improve indices of cognitive-motor function over clinically determined stimulation parameters in bilaterally implanted subthalamic DBS patients [28], without compromising motor gains based on UPDRS-III scores. Despite improvement in UPDRS-III scores with model parameters, evaluation of everyday motor function has not been included in recent investigations.

To investigate the effects of bilateral subthalamic DBS on everyday motor function, we have chosen to evaluate impairments in fine motor control using an ADL-like task to assess upper extremity function in three different stimulation conditions: clinically determined DBS parameters, patient-specific model-derived DBS parameters, and off DBS stimulation. Expanding upon the methods used in previous studies [9], [10], we have chosen to evaluate motor function in an ecological manner, by evaluating manual coordination in an ADL-like task that requires the limbs to work in together to achieve a goal [18], [29], [30].

A priori, differences between the two stimulation parameter conditions are not expected, as both conditions are programmed with the intention of alleviating motor symptoms of PD. Given that improvements in manual coordination and grip force control have been reported previously with subthalamic DBS in comparison to off stimulation states [9], [10], we hypothesize that both Clinical and Model DBS states will improve overall task performance by reducing bradykinesia (Hypothesis 1) and improving grip force production (Hypothesis 2) in PD patients compared to off stimulation states. Despite our expectation of improved grip force production, no changes in grip-load force coupling due to stimulation state are expected (Hypothesis 3), as grip-load coupling has been found to be intact in PD patients independent of treatment state [13]–[15]. It is also expected that the Clinical and Model DBS will reduce UPDRS-III motor scores compared to off DBS stimulation (Hypothesis 4). Specifically, we are interested in evaluating behavioral changes exhibited across the three stimulation states (clinical stimulation parameters, model-derived stimulation parameters, and off stimulation) in the same patient sample. By using tasks similar to activities of daily living (ADLs), our goal was to advance DBS programming and potentially improve manual function in tasks pertinent to performing daily activities.

## Methods

### Participants and Ethics Statement

Ten participants with advanced PD participated in this study (mean ± sd: age 61±8 years), Table 1 contains patient demographics. All patients underwent bilateral subthalamic DBS implantation surgery at the Cleveland Clinic at least 10 months prior to study participation. Details regarding surgical procedures for DBS implantation, and stimulation parameter programming methods, have been previously reported [28], [31]. Stimulation parameters used in clinical and model programming states can be found in Table 2. For all three sessions, patients reported the laboratory in the clinically defined OFF condition (i.e. at least 12 hours since their last dose of anti-parkinsonian medications) while on DBS with their clinically derived stimulation parameters. Following arrival at the laboratory, patient stimulators were turned off for two hours prior to testing. After the two hour off-stimulation interval, DBS stimulators were programmed according to the testing state for that session. DBS stimulators were programmed a minimum of thirty minutes prior to participation in the experimental conditions.

**Table 1:** Patient demographics, UPDRS-III scores in each of the three stimulation states, and testing session order.

				UPDRS-III Scores			
Patient	Age (yrs)	PD Duration (mo)	DBS Duration (mo)	Clinical	Model	OFF	Test Order
1	52	159	51	40	29	46	CMO
2	67	136	21	29	22	40	COM
3	73	203	57	35	29	57	COM
4	53	207	83	38	34	59	OCM
5	52	61	19	36	34	63	CMO
6	54	83	13	24	24	31	CMO
7	55	196	10	27	23	35	OMC
8	64	261	53	18	20	49	OCM
9	67	242	45	28	35	52	OCM
10	67	54	32	29	24	51	COM
*Mean*	*60*	*160*	*38*	*30*	*27*	*48*	–
*SD*	*8*	*74*	*23*	*7*	*6*	*10*	–

**Table 2:** Clinically-determined and patient-specific model-derived stimulation parameters for all patients.

Left Stimulation Parameters														
	*Clinical Settings*							*Model Settings*						
					VTA Volume							VTA Volume		
Patient	Contact	Voltage(V)	PulseWidth(µs)	Frequency(Hz)	InsideSTN(mm^3^)	OutsideSTN(mm^3^)	Total(mm^3^)	Contact	Voltage(V)	PulseWidth(µs)	Frequency(Hz)	InsideSTN(mm^3^)	OutsideSTN(mm^3^)	Total(mm^3^)
DBSDT1	1−C+	3.5	90	135	52	64	116	2−C+	2.4	60	130	21	56	77
DBSDT2	3−2+	2.9	60	130	7	18	25	3−C+	1.6	60	130	7	35	42
DBSDT3	3+1−	3.6	90	185	4	66	70	2−C+	2.4	60	130	12	40	52
DBSDT5	3+1−	3.6	90	185	28	41	69	2−C+	1.8	60	130	9	27	36
DBSDT6	2−1+	3.6	90	130	29	11	40	3−C+	2	60	130	16	35	51
DBSDT7	3−2+	3	60	130	2	23	25	3−C+	2.2	60	130	3	51	54
DBSDT8	3+2−	3.6	60	130	13	15	28	2−C+	2.4	60	130	39	40	79
DBSDT12	3+1−	3.5	90	185	34	18	52	2−C+	1.8	60	130	35	28	63
DBSDT13	2−C+	3.2	60	130	41	64	105	2−C+	2	60	130	28	40	68
DBSDT14	1−C+	3.3	60	130	45	44	89	3−C+	2.6	60	130	15	50	65
**Right Stimulation Parameters**														
	***Clinical Settings***							***Model Settings***						
					**VTA Volume**							**VTA Volume**		
**Patient**	**Contact**	**Voltage** **(V)**	**Pulse** **Width** **(µs)**	**Frequency** **(Hz)**	**Inside** **STN** **(mm^3^)**	**Outside** **STN** **(mm^3^)**	**Total** **(mm^3^)**	**Contact**	**Voltage** **(V)**	**Pulse** **Width** **(µs)**	**Frequency** **(Hz)**	**Inside** **STN** **(mm^3^)**	**Outside** **STN** **(mm^3^)**	**Total** **(mm^3^)**
DBSDT1	2−C+	3.2	60	135	52	50	102	2−C+	1.8	60	130	34	29	63
DBSDT2	3−2+	2.9	60	130	7	17	24	3−C+	2.2	60	130	14	39	53
DBSDT3	2−C+	3.6	90	185	44	94	138	3−C+	1.6	60	130	4	36	40
DBSDT5	3+1−	3.6	90	185	34	36	70	2−C+	2.2	60	130	31	16	47
DBSDT6	3+1−	3.6	90	130	20	34	54	3−C+	2.2	60	130	23	32	55
DBSDT7	3−2+	3.6	60	130	4	25	29	3−C+	2.6	60	130	9	56	65
DBSDT8	3+2−1−	3.6	60	130	0	84	84	2−C+	1.8	60	130	0	37	37
DBSDT12	3+1−	3.5	90	135	22	30	52	3−C+	2.4	60	130	14	45	59
DBSDT13	3+1−	3	60	130	29	8	37	3−C+	1.8	60	130	17	29	46
DBSDT14	2−C+	3.1	60	130	42	59	101	3−C+	2.2	60	130	10	45	55

Note that STN refers to the subthalamic nucleus.

Ethics and procedures for this study were evaluated and approved the Institutional Review Board of the Cleveland Clinic. All participants gave written informed consent according to the procedures approved by the Cleveland Clinic Institutional Review Board, in accordance with the Declaration of Helsinki. All data were collected onsite at the Cleveland Clinic, in Cleveland, Ohio, USA. None of the patients evaluated in this study exhibited cognitive issues that were significant enough to have next of kin provide informed consent. Capacity was determined via clinical cognitive evaluation using the Mini-Mental State Examination (MMSE).

### Experimental Setup and Procedure

**Figure 1:**
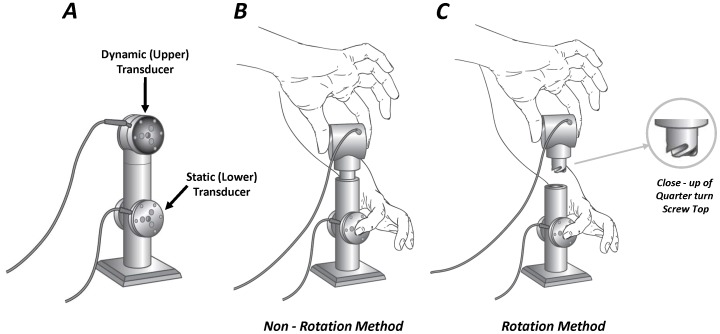
Schematics of the testing device. ***A:*** The dynamic (upper) and static (lower) transducers are indicated. ***B:*** Depiction of the task being performed with the Non-Rotation method. ***C:*** Depiction of the task being performed with the Rotation method.

A two-transducer system (Figure 1), was used to determine the time and force characteristics of bimanual tasks. The tasks involved connecting two independent objects together using one of two movements: (a) placing one object on top of another, or (b) connecting the two objects by rotating the upper object while stabilizing the lower object. Grip and load forces exerted by the hands were recorded simultaneously using force-moment transducers (Mini-40 transducers; ATI Industrial Automation, Garner, NC, USA). Two different articulation types (simple cylinder and a quarter turn screw top, referred to as *Non-Rotation* and *Rotation* tasks, respectively) between the two sensors were used throughout each testing session (Panels B and C of Figure 1).

Participants were instructed to perform each task using only a pinch grip. Both the upper (dynamic) and lower (static) objects were freely moveable; however, the location and orientation of the static object was prescribed. Subjects were not permitted to move the static object during testing. Any trial in which movement of the static object occurred was omitted.

Overall, eight different bimanual configurations were performed in each testing session. Five trials were collected in each of the eight tested configurations (40 total trials in each session, 120 trials total). Details regarding each of the tested conditions can be found explicitly described in [18], [29], [30]. Briefly, each bimanual task involved either the connecting of two separate objects into one object (Connect) or disconnecting an object into two separate objects (Disconnect). This action was performed using rotational (Rotation) and non-rotational (Non-rotation) actions of the upper/dynamic transducer.

No contact of either transducer was permitted prior to trial onset; subjects were instructed to begin each trial with both hands placed palm down on the surface of the table. Up to three practice trials were offered to each subject prior to the onset of data collection for each condition. On average, each subject performed one practice trial. In each of the three sessions, the presentation of conditions was block randomized for each subject; articulation type was used as the blocking factor.

Data were collected during three visits to the Neural Control Laboratory at the Cleveland Clinic. Each visit was separated by a minimum of 72 hours. Each participant completed the experiment under three different stimulation conditions: clinically-determined DBS parameters (Clinical), patient-specific model-derived DBS parameters (Model), and off DBS stimulation (Off).

### DBS Conditions

The clinically-determined DBS stimulation parameters used in this study were the settings derived for each patient via standard clinical care. These settings were stable for at least 6 months prior to study participation. The clinical programming of stimulators was overseen by an experienced DBS programming team consisting of a programming nurse and a movement disorders neurologist specializing in Parkinson’s disease.

Model derived stimulation parameters were defined from a patient-specific DBS computer model of each side of the patient’s brain created in Cicerone v1.2, a freely available academic DBS research tool [32]. The details of the model development and stimulation parameter selection process are described in [28]. Briefly, each patient-specific DBS model included coupled integration of MRI/CT data, intra-operative electrophysiology data, 3D brain atlas surfaces, DBS electrode models, and volume of tissue activation (VTA) predictions all co-registered into the neurosurgical stereotactic coordinate system.

Based on previous experience developing patient-specific models of therapeutic subthalamic DBS [26], [33], a theoretical ellipsoid target volume was defined that encompassed the dorsal subthalamic nucleus and the white matter dorsal to the subthalamic nucleus [28]. We defined a stimulation parameter setting for each side of each patient’s brain that maximized stimulation coverage of the target volume and minimized stimulation spread outside of the target volume. These model settings were defined without knowledge of the clinical stimulation settings or clinical programming notes, and based solely on customizing the stimulation to the theoretical target volume in each patient.

Model settings were defined using theoretical predictions of the VTA. The VTA provides an electrical prediction of the volume of axonal tissue directly activated by DBS for a given stimulation parameter setting. The software provided the ability to interactively visualize and evaluate a wide range of stimulation parameter settings and calculate the overlap of the VTA with the brain anatomy and target volume. Following completion of the experimental testing we used Cicerone to quantify the VTAs for each patient under both the Model and Clinical settings, along with their respective overlap with the subthalamic nucleus atlas volume available in the software.

### Data Analysis

Grip and load forces were analyzed several different ways in this experiment. At the most basic level of analysis, the maximum magnitudes of the exerted forces were evaluated, as well as the rate of grip force production (*dF^G^/dt*) in each of the tested conditions. Total grip and load forces were considered twice the measured value as only two force-torque sensors were used in the experimental setup. Correlation between grip and load forces within each hand was computed to evaluate within-limb control during task performance. Data analysis algorithms related to the analysis of raw force-torque data signals, data filtering techniques, and movement onset and termination identification were based on previous work [18], [29], [30].

### Statistics

The data are presented in the text and figures as means and standard errors. Analyses of variance (ANOVAs) were performed on the data with the factors of: *Method* (two levels; Rotation and Non-Rotation), and *Task* (two levels; Connect and Disconnect), *Stimulation* (three levels; Clinical, Model, and Off), and *Transducer* (two levels; Static and Dynamic). Evaluation of conditions appropriate for parametric analyses were performed prior to statistical analyses. For all ANOVAs, the assumption of sphericity was verified using Mauchly’s sphericity test. If sphericity was violated, the degrees of freedom were adjusted as necessary using Greenhouse-Geisser corrections. Correlation coefficient (*r*) values from the regression analysis were subjected to Fisher z-transformation to mitigate the ceiling effects inherent to these variables. Non-transformed data are presented in the figures to avoid confusion. Post-hoc pairwise comparisons to elucidate differences among *Stimulation* (Clinical, Model, and Off) state were performed with Bonferroni corrections were used to evaluate significant effects of the ANOVA analyses. Data regarding VTA measurements were found to obey normal distributions and were therefore evaluated via paired t-tests between on-stimulation states (Clinical vs Model). Non-parametric analyses were used in the evaluation of UPDRS-III scores. Kruskal-Wallis analyses were used to determine if differences existed in UPDRS scores across the three stimulation states. Mann-Whitney tests were then used to determine differences in UPRS-III scores between stimulation states (Clinical vs Model, Clinical vs Off, and Model vs Off). All ANOVAs were performed using SPSS; all non-parametric analyses were performed using Minitab, due to ease of usability of the interface for non-parametric analyses.

## Results

### UPDRS-III Scores

**Figure 2:**
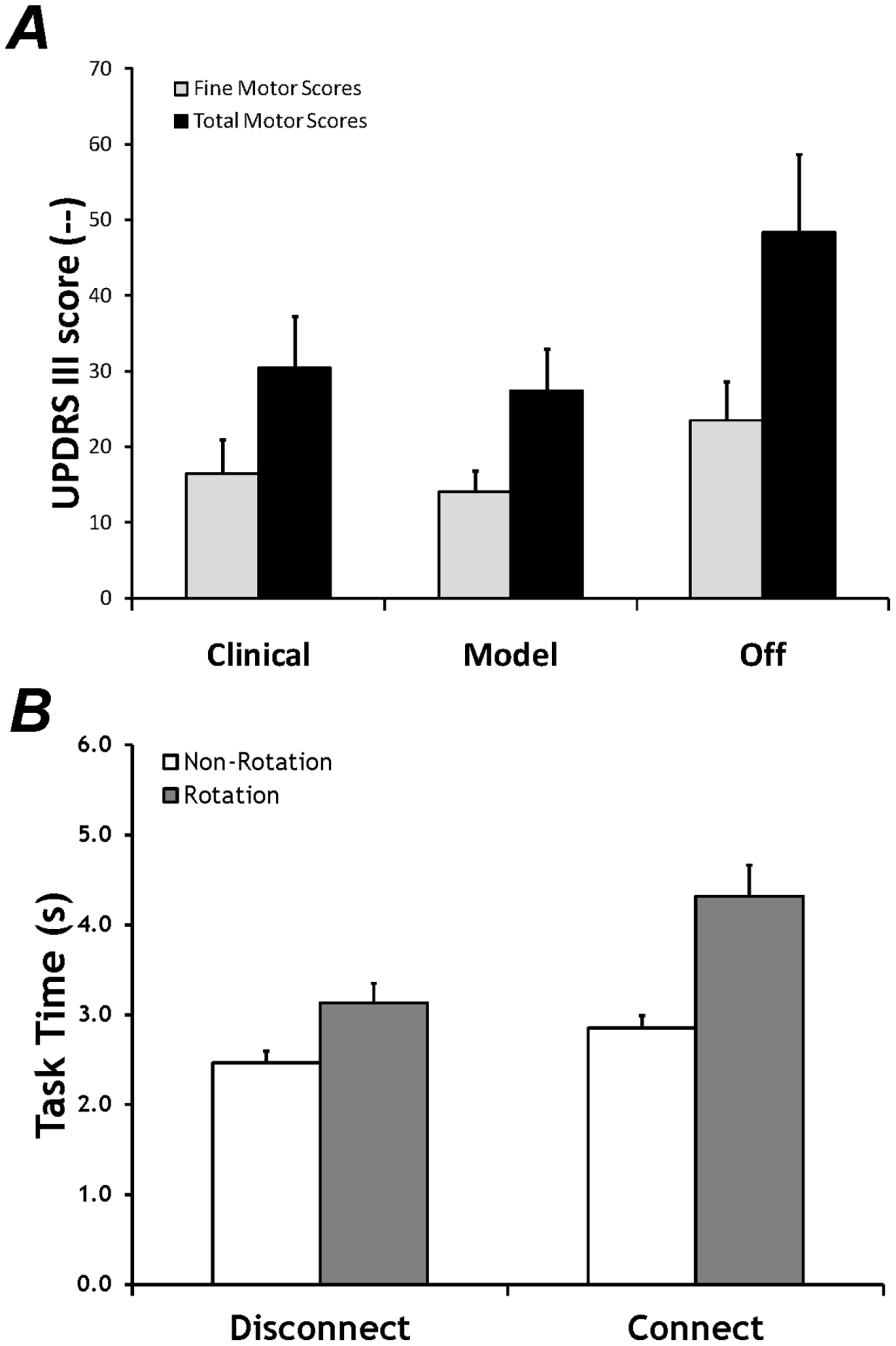
Mean and standard error UPDRS-III scores and task time. ***A:*** Fine and total UPDRS-III scores. Significant reductions in both the fine and total UPDRS-III scores were found for the on-stimulation states as compared to the Off DBS state. ***B:*** Task time, averaged across stimulation state. A significant decrease in task time in Non-Rotational actions and in Disconnect-type tasks were found as compared to Rotational actions and Connect-type tasks.

UPDRS-III scores for each of the DBS states revealed differences between the on-DBS states versus the off-DBS state (K = 16.04, p<0.001). Both clinically-determined and model-derived parameters were associated with lower overall UPDRS-III scores as compared to off-stimulation (Clinical vs Off: W = 147.0, p<0.005; Model vs Off: W = 151.1, p<0.001), see Figure 2A. No difference between the on-stimulation states was detected (Clinical vs Model: W = 120.5, p = 0.26). UPDRS-III scores associated with hand function (hand resting and action tremor, upper extremity rigidity, finger taps, hand grips, and hand pronation/supination) also revealed lower scores for on-DBS states versus the off-DBS state (K = 12.93, p<0.001). Similar to the overall UPDRS-III scores, no difference between on-DBS states were detected (Clinical vs Model: W = 122.0, p = 0.21)., while both clinically-determined and model-derived parameters were associated with lower scores as compared to off-stimulation (Clinical vs Off: W = 139.5, p<0.01; Model vs Off: W = 148.0, p<0.005), also shown in Figure 2A.

### Task Time

The overall time to perform the bimanual tasks (task time) was not different across DBS conditions. Similar to analysis of healthy young individuals [29] and healthy older adults [30], task time was affected by the *Task* and *Method*. Task time was greater when the two objects were being connected to each other (*Task:* F_1,68_ = 14.2, p<0.001) and during rotational actions (*Method:* F_1,68_ = 25.8, p<0.001), illustrated in Figure 2B. While task time increased when the two objects were being connected together; this effect was particularly striking when rotational actions were used. No interaction effects were found.

### Kinetics

#### Applied Forces

**Figure 3:**
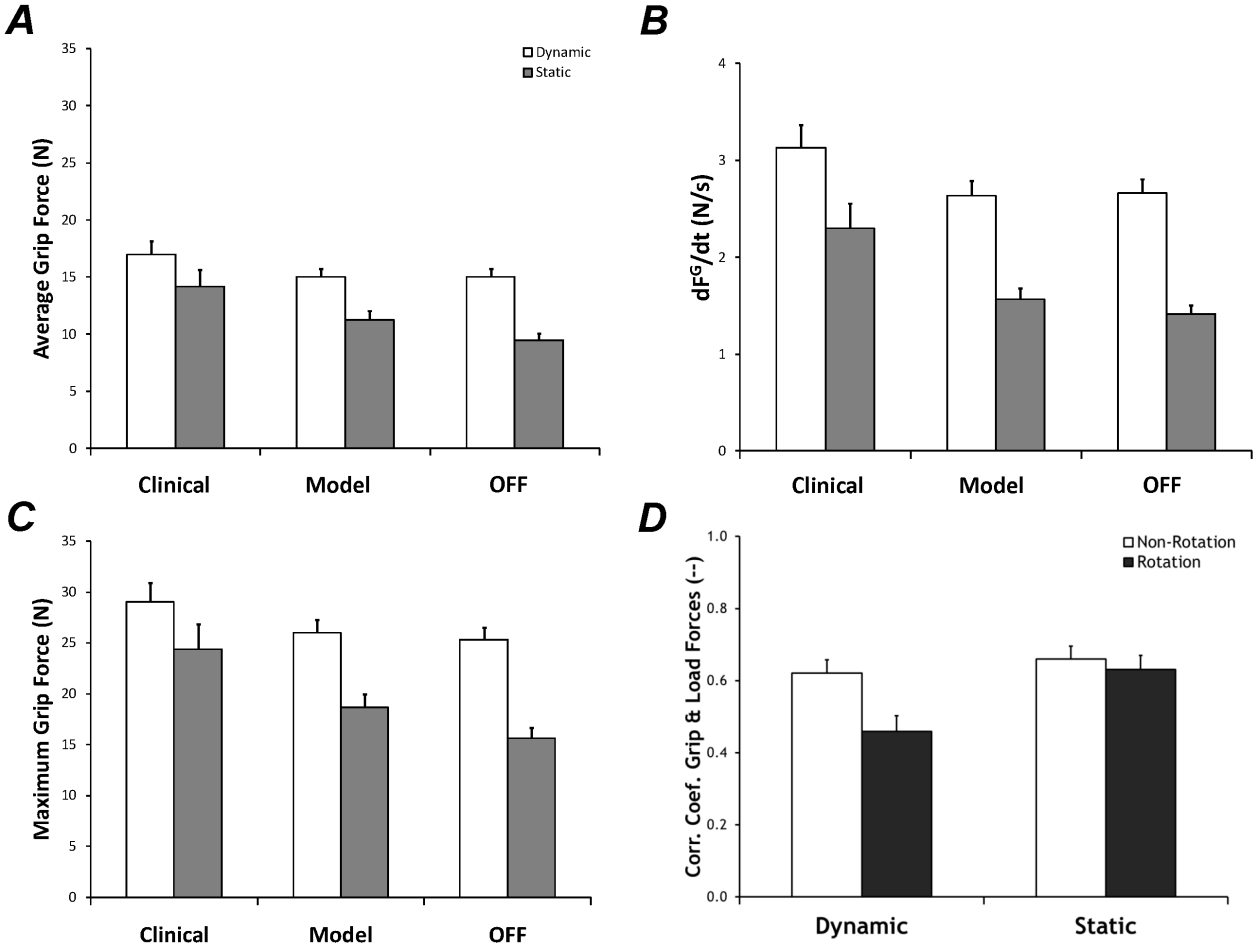
Mean and standard error of force production. Significant differences in the forces exerted on the two transducers (Dynamic vs Static) are shown in each panel. Significant differences among the three DBS parameter conditions can be found in panels A-C, whereas a difference between transducers is shown in panel D. ***A:*** Average grip force. ***B:*** Rate of grip force production. ***C:*** Maximum grip force. ***D:*** Within-hand grip-load force correlation.

Across all conditions, the average grip force produced was 11.6±0.9 N and 15.7±1.0 N for the static and dynamic transducers, respectively. Average grip force was affected by DBS stimulation state and transducer, as shown in Figure 3A. Average grip force production exhibited by participants during testing with Clinical DBS parameters was significantly greater compared to the Off DBS condition (*Stimulation*: F_1.3,134.6_ = 7.4, p<0.005), confirmed by post-hoc analysis. Average grip force during testing with Model DBS parameters was not significantly different from Clinical or Off DBS conditions. Average grip forces were also found to be greater when exerted on the dynamic transducer (*Transducer*: F_1.0,134.6_ = 49.6, p<0.001). The disproportion of applied grip forces between the two transducers was most notable during the Off DBS stimulation condition. This finding was confirmed by the presence of a *Stimulation x Transducer* interaction (F_1.98,134.6_ = 4.9, p<0.01), illustrated in Figure 3A.

Similar to the average grip force findings, maximum grip force was significantly larger on the dynamic transducer (*Transducer:* F_1.0,122.9_ = 61.6, p<0.001) and during the Clinical DBS condition (*Stimulation*: F_1.4,122.9_ = 10.2, p<0.001). Maximal grip force was largest during the Clinical DBS condition as compared to the Off DBS condition, whereas the Model DBS condition did not differ from the other two stimulation states, confirmed via post-hoc. An imbalance in applied maximum applied grip force between the two transducers was most notable during the Off DBS state, such that larger maximal grip forces were applied to the dynamic transducer, confirmed by the *Stimulation x Transducer* interaction (F_1.8,122.9_ = 7.1, p<0.005), illustrated in Figure 3C.

The rate of grip force production (dFG/dt) exhibited similar trends to average and maximal grip force production, such that dFG/dt was significantly larger on the dynamic transducer (*Transducer:* F_1.0,118.6_ = 133.1, p<0.001) and when participants were experiencing the Clinical DBS condition (*Stimulation*: F_1.3,32.3_ = 9.8, p<0.001); however, no interaction effects were found, as shown in Figure 3B. Post hoc analysis indicated that during the Clinical DBS condition, dFG/dt was significantly larger than dFG/dt associated with the Model DBS and Off DBS conditions.

#### Kinetic Coupling

In this analysis of within-hand coordination, the kinetic coupling measures did not show effects of stimulation state. Despite lack of a stimulation effect, within-hand grip-load force correlation was larger for the static transducer (*Transducer:* F_1.0,135.1_ = 25.5, p<0.001) and in non-rotation tasks (*Method:* F_1,68_ = 12.0, p<0.001), shown in Figure 3D. The difference between the grip-load correlation during use of the rotation and non-rotation methods was most pronounced on the dynamic transducer, confirmed by the *Transducer x Method* interaction (F_1.0,135.1_ = 4.9, p<0.05).

#### Volume of Tissue Activated by DBS

The VTAs for the Clinical and Model DBS are located in Table 3. The total VTA and VTA outside the subthalamic nucleus were not affected by stimulation setting in this patient population; however, the VTA inside the subthalamic nucleus was found to be largest in the Clinical settings (t_19_ = 2.28, p<0.05).

**Table 3:** Mean (SD) volume of tissue activated by the clinically-determined and patient-specific model-derived stimulation parameters, averaged across all patients.

	ClinicalSettings	ModelSettings	*Signif.*
Inside STN Volume (mm^3^)	**25.5 (17.5)**	**17.1 (11.3)**	***p<.05***
Outside STN Volume (mm^3^)	40.1 (24.9)	38.3 (10.3)	–
Total VTA (mm^3^)	65.5 (34.5)	55.4 (12.2)	–

Note that STN refers to the subthalamic nucleus.

## Discussion

In this investigation of the effects of DBS on fine motor control, we hypothesized DBS would improve basic features of bimanual dexterity. Specifically, we hypothesized that testing clinically-determined and patient-specific model-derived DBS parameters would be associated with reduced bradykinesia and improved grip force production (Hypotheses 1 and 2, respectively). With respect to these hypotheses, analysis of the current data set did not reveal significant DBS-related improvements in bradykinesia during bimanual tasks; however, stimulation condition did affect the magnitude of grip forces exerted. Consistent with our third hypothesis, grip-load coupling was found to be intact in PD patients independent of treatment state. As per our fourth hypothesis, we expected both clinically-determined and model-derived DBS stimulation conditions to be associated with lower overall UPDRS-III motor scores as compared to the off-stimulation condition. Despite the limited stimulation effects on bimanual actions, significant differences in UPDRS-III scores were found. In the following paragraphs, we address the differential effects of DBS condition on motor function as well as the physiological and programming implications of DBS.

### Task Performance and Effects of Stimulation State

Regarding our hypotheses, a prominent feature of PD movement, bradykinesia (measured by task time), was not different across stimulation conditions, contrary to earlier reports [34]. The lack of comparative bradykinetic hand action across stimulation states may be due to the convergence of several factors. The current study investigated bilaterally implanted DBS patients, whereas earlier studies only investigated unilaterally implanted DBS patients [34]. Symmetry in DBS implantation may have functional implications, such that modified neural output of the basal ganglia from both hemispheres may be used to improve rhythmic activities, such as gait, but not discrete movements [34], [35]. The lack of improved bradykinesia may also be due to the tasks being performed at self-selected speeds. Movement slowness emerges when PD patients are required to perform tasks at maximal speeds [36], [37]; whereas the tasks in this study were performed at self-selected speeds, consistent with performance of activities of daily living. Performance of daily tasks such as bathing, grooming, and feeding typically occur at self-selected speeds; suggesting that bradykinesia in such tasks may not be alleviated by DBS implantation and stimulation. In contrast, the lack of stimulation effects on grip-load coupling is consistent with PD patients retaining the ability to coordinate forces exerted on hand-held objects despite difficulty in other measures of motor function [13]–[15].

Despite these findings, differences in grip force production due to stimulation state were exhibited. Abnormal grip force production has been reported with PD [13], [15]; however, discrepancies in the effect of DBS grip force production in PD have emerged [38], [39]. Consistent with earlier reports [39], clinically-determined DBS parameters were associated with increased grip force magnitude and production rates compared to the off-stimulation state. Grip data collected during the patient-specific model-derived DBS parameters condition was not significantly different from either the clinically-determined DBS condition or the off-stimulation condition, suggesting that specific aspects of these parameters may limit improvement in fine motor function despite overall improvements in gross motor function.

### Possible Physiological Mechanisms and Implications for DBS Programming

In the current study, clinically-determined and patient-specific model-derived DBS parameters both produced improved clinical rating scores. However, patient-specific model-derived DBS parameters do not appear to be as effective in improving motor performance in ADL-like tasks compared to clinically-determined DBS parameters. While previous work has shown equivalence in motor performance between the two parameter groups under dual-task conditions (i.e. simultaneous performance of a cognitive and motor task) [28], improvement with patient-specific model-derived DBS parameters has been associated with smaller VTAs due to model-based volume prediction, and, theoretically minimal spread of current to non-motor regions of the subthalamic nucleus. Consistent with [28], smaller VTAs within the subthalamic nucleus were found in the current data set with patient-specific model-derived DBS parameters as compared to clinically-determined parameters. As the general target of stimulation for subthalamic DBS is the dorsolateral subthalamic nucleus [40], smaller activation volumes of this motor area in the subthalamic nucleus with model-derived parameters would lead to more localized neural stimulation within the subthalamic nucleus. Given the somatotopic organization of the subthalamic nucleus [41]–[43], it is likely that the current generated according to the model-derived parameters reached neurons associated with axial and proximal anatomic structures. The representation of axial and proximal limb areas have been found to be larger than that of distal limb areas within the subthalamic nucleus [41], [44]–[46]. The smaller VTAs associated with model-derived parameters may not reach the less dense and more laterally located neurons associated with fine coordination of distal musculature, such as the neurons associated with finger and hand actions, producing a potential trade-off in cognitive-motor function with DBS. Future work in optimizing model-derived VTAs to affect only motor regions of the subthalamic nucleus may alleviate this trade-off by improving cognitive function while not compromising motor function in implanted DBS patients. This may be partially accomplished by utilizing measures of fine motor action performed by the distal musculature as a means to evaluate motor performance in the process of selecting the patient-specific model-derived DBS parameters. This would represent a hybrid approach to DBS programming where the patient-specific model-derived parameters define the initial starting point, while clinical input and quantitative data are used to establish final settings [18].

## References

[1] Deep-Brain Stimulation of the Subthalamic Nucleus or the Pars Interna of the Globus Pallidus in Parkinson’s Disease (2001) New England Journal of Medicine. 345: 956–963 10.1056/NEJMoa000827 11575287

[2] Chung SJ, Jeon SR, Kim SR, Sung YH, Lee MC (2006) Bilateral Effects of Unilateral Subthalamic Nucleus Deep Brain Stimulation in Advanced Parkinson’s Disease. Eur Neurol 56: 127–132 10.1159/000095704 16960454

[3] Kumar R, Lozano AM, Sime E, Halket E, Lang AE (1999) Comparative effects of unilateral and bilateral subthalamic nucleus deep brain stimulation. Neurology 53: 561. 10449121 10.1212/wnl.53.3.561

[4] Volkmann J, Allert N, Voges J, Sturm V, Schnitzler A, et al (2004) Long-term results of bilateral pallidal stimulation in Parkinson’s disease. Ann Neurol 55: 871–875 10.1002/ana.20091 15174022

[5] Nowak DA, Topka H, Tisch S, Hariz M, Limousin P, et al (2005) The beneficial effects of subthalamic nucleus stimulation on manipulative finger force control in Parkinson’s disease. Experimental neurology 193: 427–436. 15869945 10.1016/j.expneurol.2005.01.003

[6] Sturman MM, Vaillancourt DE, Verhagen Metman L, Bakay RAE, Corcos DM (2010) Effects of five years of chronic STN stimulation on muscle strength and movement speed. Experimental Brain Research 205: 435–443. 20697699 10.1007/s00221-010-2370-8

[7] Vaillancourt DE, Prodoehl J, Sturman MM, Bakay RAE, Metman LV, et al (2006) Effects of deep brain stimulation and medication on strength, bradykinesia, and electromyographic patterns of the ankle joint in Parkinson’s disease. Movement Disorders 21: 50–58. 16124011 10.1002/mds.20672 PMC2373255

[8] Vaillancourt DE, Mayka MA, Thulborn KR, Corcos DM (2004) Subthalamic nucleus and internal globus pallidus scale with the rate of change of force production in humans. Neuroimage 23: 175–186. 15325364 10.1016/j.neuroimage.2004.04.040

[9] Alberts JL, Okun MS, Vitek JL (2008) The persistent effects of unilateral pallidal and subthalamic deep brain stimulation on force control in advanced Parkinson’s patients. Parkinsonism & related disorders 14: 481–488. 18342565 10.1016/j.parkreldis.2007.11.014 PMC2605295

[10] Alberts JL, Voelcker-Rehage C, Hallahan K, Vitek M, Bamzai R, et al (2008) Bilateral subthalamic stimulation impairs cognitive–motor performance in Parkinson’s disease patients. Brain 131: 3348. 18842609 10.1093/brain/awn238 PMC2639204

[11] Vrancken AMPM, Allum JHJ, Peller M, Visser JE, Esselink RAJ, et al (2005) Effect of bilateral subthalamic nucleus stimulation on balance and finger control in Parkinson’s disease. J Neurol 252: 1487–1494 10.1007/s00415-005-0896-7 16021354

[12] Fellows SJ, Noth J (2004) Grip force abnormalities in de novo Parkinson’s disease. Movement Disorders 19: 560–565. 15133821 10.1002/mds.10710

[13] Fellows SJ, Noth J, Schwarz M (1998) Precision grip and Parkinson’s disease. Brain 121: 1771–1784 10.1093/brain/121.9.1771 9762964

[14] Gordon AM, Ingvarsson PE, Forssberg H (1997) Anticipatory Control of Manipulative Forces in Parkinson’s Disease. Experimental neurology 145: 477–488. 9217084 10.1006/exnr.1997.6479

[15] Ingvarsson PE, Gordon AM, Forssberg H (1997) Coordination of Manipulative Forces in Parkinson’s Disease. Experimental neurology 145: 489–501. 9217085 10.1006/exnr.1997.6480

[16] Nowak DA, Hermsdörfer J (2002) Coordination of Grip and Load Forces During Vertical Point-to-Point Movements With a Grasped Object in Parkinson’s Disease. Behavioral Neuroscience 116: 837–850. 12369804

[17] Pradhan SD, Brewer BR, Carvell GE, Sparto PJ, Delitto A, et al (2010) Assessment of Fine Motor Control in Individuals with Parkinson’s Disease Using Force Tracking with a Secondary Cognitive Task. Journal of Neurologic Physical Therapy 34: 32. 20212366 10.1097/NPT.0b013e3181d055a6

[18] Gorniak SL, Machado AG, Alberts JL (2013) Force coordination during bimanual task performance in Parkinson’s disease. Exp Brain Res 229: 261–271 10.1007/s00221-013-3608-z 23811728 PMC10103102

[19] Lazarus A, Stelmach GE (1992) Interlimb coordination in Parkinson’s disease. Mov Disord 7: 159–170 10.1002/mds.870070211 1584238

[20] Behari M, Srivastava AK, Pandey RM (2005) Quality of life in patients with Parkinson’s disease. Parkinsonism & Related Disorders 11: 221–226. 15878582 10.1016/j.parkreldis.2004.12.005

[21] Rahman S, Griffin HJ, Quinn NP, Jahanshahi M (2008) Quality of life in Parkinson’s disease: The relative importance of the symptoms. Mov Disord 23: 1428–1434 10.1002/mds.21667 18543333

[22] Mera T, Vitek JL, Alberts JL, Giuffrida JP (2011) Kinematic optimization of deep brain stimulation across multiple motor symptoms in Parkinson’s disease. Journal of Neuroscience Methods. Available: http://linkinghub.elsevier.com/retrieve/pii/S0165027011001671. Accessed 6 June 2011. 10.1016/j.jneumeth.2011.03.019 PMC3122330 21459111

[23] Goetz CG, Fahn S, Martinez-Martin P, Poewe W, Sampaio C, et al (2007) Movement Disorder Society-sponsored revision of the Unified Parkinson’s Disease Rating Scale (MDS-UPDRS): Process, format, and clinimetric testing plan. Mov Disord 22: 41–47 10.1002/mds.21198 17115387

[24] Freund H-J (2005) Long-term effects of Deep Brain Stimulation in Parkinson’s Disease. Brain 128: 2222–2223 10.1093/brain/awh634 16183664

[25] Saint-Cyr JA, Trépanier LL, Kumar R, Lozano AM, Lang AE (2000) Neuropsychological consequences of chronic bilateral stimulation of the subthalamic nucleus in Parkinson’s disease. Brain 123: 2091–2108 10.1093/brain/123.10.2091 11004126

[26] Butson CR, Cooper SE, Henderson JM, McIntyre CC (2007) Patient-specific analysis of the volume of tissue activated during deep brain stimulation. Neuroimage 34: 661–670. 17113789 10.1016/j.neuroimage.2006.09.034 PMC1794656

[27] McIntyre CC, Miocinovic S, Butson CR (2007) Computational analysis of deep brain stimulation. Expert Rev Med Devices 4: 615–622 10.1586/17434440.4.5.615 17850196

[28] Frankemolle AM, Wu J, Noecker AM, Voelcker-Rehage C, Ho JC, et al (2010) Reversing cognitive–motor impairments in Parkinson’s disease patients using a computational modelling approach to deep brain stimulation programming. Brain 133: 746. 20061324 10.1093/brain/awp315 PMC2842509

[29] Gorniak SL, Alberts JL (2013) Effects of task complexity on grip-to-load coordination in bimanual actions. Experimental Brain Research 225: 559–567 10.1007/s00221-012-3395-y 23307159 PMC10103104

[30] Gorniak SL, Alberts JL (2013) Effects of aging on force coordination in bimanual task performance. Exp Brain Res. doi:10.1007/s00221-013-3644-8. 10.1007/s00221-013-3644-8 PMC10103123 23852325

[31] Machado A, Rezai AR, Kopell BH, Gross RE, Sharan AD, et al (2006) Deep brain stimulation for Parkinson’s disease: surgical technique and perioperative management. Mov Disord 21 Suppl 14S247–258 10.1002/mds.20959 16810722

[32] Miocinovic S, Noecker AM, Maks CB, Butson CR, McIntyre CC (2007) Cicerone: stereotactic neurophysiological recording and deep brain stimulation electrode placement software system. Acta Neurochir Suppl 97: 561–567. 10.1007/978-3-211-33081-4_65 17691348

[33] Maks CB, Butson CR, Walter BL, Vitek JL, McIntyre CC (2009) Deep brain stimulation activation volumes and their association with neurophysiological mapping and therapeutic outcomes. Journal of Neurology, Neurosurgery & Psychiatry 80: 659–666 10.1136/jnnp.2007.126219 PMC2859444 18403440

[34] Alberts JL, Elder CM, Okum MS, Vitek JL (2004) Comparison of pallidal and subthalamic stimulation on force control in patient’s with Parkinson’s disease. Motor Control 8: 484–499. 15585903 10.1123/mcj.8.4.484

[35] Bastian AJ, Kelly VE, Revilla FJ, Perlmutter JS, Mink JW (2003) Different effects of unilateral versus bilateral subthalamic nucleus stimulation on walking and reaching in Parkinson’s disease. Mov Disord 18: 1000–1007 10.1002/mds.10493 14502667

[36] Gordon AM (1997) Object release in patients with Parkinson’s disease. Neurosci Lett 232: 1–4. 9292877 10.1016/s0304-3940(97)00556-9

[37] Gordon AM (1998) Task-dependent deficits during object release in Parkinson’s disease. Exp Neurol 153: 287–298 10.1006/exnr.1998.6880 9784288

[38] Wenzelburger R, Kopper F, Zhang B-R, Witt K, Hamel W, et al (2003) Subthalamic nucleus stimulation for Parkinson’s disease preferentially improves akinesia of proximal arm movements compared to finger movements. Movement Disorders 18: 1162–1169 10.1002/mds.10501 14534921

[39] Fellows SJ, Kronenbürger M, Allert N, Coenen VA, Fromm C, et al (2006) The effect of subthalamic nucleus deep brain stimulation on precision grip abnormalities in Parkinson’s disease. Parkinsonism & Related Disorders 12: 149–154. 16549385 10.1016/j.parkreldis.2005.12.001

[40] Rodriguez-Oroz MC, Rodriguez M, Guridi J, Mewes K, Chockkman V, et al (2001) The subthalamic nucleus in Parkinson’s disease: somatotopic organization and physiological characteristics. Brain 124: 1777–1790 10.1093/brain/124.9.1777 11522580

[41] DeLong MR, Crutcher MD, Georgopoulos AP (1985) Primate globus pallidus and subthalamic nucleus: functional organization. Journal of Neurophysiology 53: 530–543. 3981228 10.1152/jn.1985.53.2.530

[42] Wichmann T, Bergman H, DeLong MR (1994) The primate subthalamic nucleus. III. Changes in motor behavior and neuronal activity in the internal pallidum induced by subthalamic inactivation in the MPTP model of parkinsonism. Journal of Neurophysiology 72: 521–530. 7983516 10.1152/jn.1994.72.2.521

[43] Nambu A, Takada M, Inase M, Tokuno H (1996) Dual somatotopical representations in the primate subthalamic nucleus: evidence for ordered but reversed body-map transformations from the primary motor cortex and the supplementary motor area. The Journal of Neuroscience 16: 2671–2683. 8786443 10.1523/JNEUROSCI.16-08-02671.1996 PMC6578767

[44] Wichmann T, Bergman H, DeLong MR (1994) The primate subthalamic nucleus. I. Functional properties in intact animals. J Neurophysiol 72: 494–506. 7983514 10.1152/jn.1994.72.2.494

[45] Miyachi S, Lu X, Imanishi M, Sawada K, Nambu A, et al (2006) Somatotopically arranged inputs from putamen and subthalamic nucleus to primary motor cortex. Neuroscience Research 56: 300–308. 16973231 10.1016/j.neures.2006.07.012

[46] Romanelli P, Esposito V, Schaal DW, Heit G (2005) Somatotopy in the basal ganglia: experimental and clinical evidence for segregated sensorimotor channels. Brain Research Reviews 48: 112–128 10.1016/j.brainresrev.2004.09.008 15708631

